# Monitoring of *Campylobacter jejuni* in a chicken infection model by measuring specific volatile organic compounds and by qPCR

**DOI:** 10.1038/s41598-022-15863-7

**Published:** 2022-07-11

**Authors:** Julia Hankel, Timothy Gibson, Julia Skov, Karsten Brandt Andersen, Michelle Dargatz, Andreas Kappel, Frank Thiemann, Ben Curtis, Bussarakam Chuppava, Christian Visscher

**Affiliations:** 1 Institute for Animal Nutrition, University of Veterinary Medicine Hannover, Foundation, Bischofsholer Damm 15, 30173 Hannover, Germany; 2 RoboScientific Ltd, Espace North, 181 Wisbech Road, Littleport, CB6 1RA Cambridgeshire UK; 3 AeroCollect A/S, Park Alle 345, 2605 Brøndby, Denmark; 4 Evonik Operations GmbH, Nutrition & Care, Rodenbacher Chaussee 4, 63457 Hanau-Wolfgang, Germany

**Keywords:** Animal biotechnology, Biotechnology, Computational biology and bioinformatics, Microbiology, Diseases

## Abstract

*Campylobacter* is one of the leading bacterial foodborne pathogens worldwide. Poultry is the host species with this pathogen with the highest clinical impact. Flocks become colonised with *Campylobacter*, which leads to contamination of product entering the food-chain. Rapid and reliable *Campylobacter* detection methods could support controls to minimize the risks of contamination within the food-chain, which would easier enable the implementation of a logistical slaughter schedule or other control options. The present study evaluates current and emerging *C.* *jejuni* detection technologies on air samples in a unique study set-up of pre-defined *C.* *jejuni* prevalences. Both non-invasive detection technologies on air samples by subsequent measuring of volatile organic compounds (VOCs) or by qPCR detected the *C.* *jejuni* presence and could additionally distinguish between the number of present *C.* *jejuni*-positive birds in the study set-up. Nevertheless, electrostatic air samplers diagnosed fewer birds as *C.* *jejuni*-positive compared to the cultivation-based method. By measuring the VOCs, it was possible to detect the presence of two positive birds in the room. This apparent high sensitivity still needs to be verified in field studies. Techniques, such as these promising methods, that can facilitate *C.* *jejuni* surveillance in poultry flocks are desirable to reduce the risk of infection for humans.

## Introduction

The genus *Campylobacter* comprises a large and diverse group of bacteria, with currently 26 species, being one of the leading bacterial foodborne pathogens worldwide^1,2^. Among the *Campylobacter* species, *Campylobacter jejuni* has the highest clinical impact^3,4^, accounting for over 80% of *Campylobacter* infections^5^. *C. jejuni* is sensitive to normal oxygen concentrations and also susceptible to heating, freezing and acidity^5^. Infection with *Campylobacter* is quite common and causes significant healthcare and societal costs^6^. The infection usually manifests as an acute gastroenteritis^7^, but it can also lead to chronic sequelae, arthritis, haemolytic uraemic syndrome (HUS), inflammatory bowel disease and functional gastrointestinal disorders, and in a severe form to Guillain–Barré syndrome^6,7^. The incidence of infections with *Campylobacter* was estimated as 96 million per year on a global scale^8^, with an incidence of 14.3 infections per 100,000 inhabitants, and an associated cost of $1.7 billion in the United States alone^9^.

The most relevant reservoir of *C. jejuni* is poultry, and colonisation of broiler flocks on farms often leads to contamination along the poultry production chain, leading to contamination of poultry meat at retail^10,11^. The European Food Safety Authority (EFSA) has estimated that chicken meat consumption accounts for 20–30% of campylobacteriosis in the EU, whereas the remaining cases may be attributed to the chicken reservoir as a whole^12,13^. This alone underlines the importance of broiler meat production in particular and poultry farming in general, as the main cause of campylobacteriosis.

The reduction in *Campylobacter* levels in the barn, and even more importantly in the broiler intestines, is an effective measure to prevent contamination of poultry meat during slaughtering and meat production^10,13,14^*.* Consequently, the reduction in *Campylobacter* concentration in the broiler intestines at slaughter by > 3 log_10_ units has been estimated to lead to a reduction in risk of human infection by at least 90%^12^. It is therefore vital to decrease *Campylobacter* load at all stages of production by effective intervention strategies. Testing methods to support in-line testing could support and inform effective intervention strategies. The contamination of non-infected batches of chickens depends on the *Campylobacter*-positive status of previously slaughtered batches and the amount of cross-contamination, while cross-contamination can occur in the course of a day and the implementation of logistic or scheduled slaughter could preserve *Campylobacter*-free batches or enable subjecting carcasses from these flocks to specific treatment^13,15,16^. Several intervention strategies to control the occurrence of *Campylobacter* on farm exist, including hygiene barriers and access restriction, but also treatment of flocks with probiotics, phages or organic acids^10,17^. The prerequisite is knowledge about the *Campylobacter* status of the delivered flocks, which requires specific and rapid testing shortest time before slaughter^15^.

The presence of *Campylobacter* in poultry barns, but also contamination of poultry meat and carcasses with *Campylobacter*, is routinely detected and quantified by well-established methods such as microbiological characterisation, immunological methods and nucleic acids amplification technologies^1,13^. More recently, methods based on quantitative real-time Polymerase Chain reaction (qPCR), droplet PCR (dPCR), Next Generation Sequencing (NGS), microarray and biosensor technologies have been introduced^18,19^, but these methods are not yet used in routine production. All these detection methods are typically used to analyse poultry meat or carcasses for the presence of *Campylobacter*, as this is the most relevant sample type to determine the hazard potential for humans. Even if those new detection technologies require development and further investigation, they can also be applied to detect *Campylobacter* colonization in on-farm monitoring^19^.

More recently, poultry barn air has been introduced as an additional sample matrix for detecting *Campylobacter* at production level by PCR^19–22^. In these methods, *Campylobacter* present in barn air dust is captured, for example, through filters, and then detected by PCR. These methods have the advantage over analysing faecal material that the sample collection step is faster and more standardised, and there is also a potential for automation and continuous measurements. Moreover, air dust samples have the advantage that the sample is more representative for the flock when compared to faecal samples that typically only contain material from a manageable number of birds. Another attractive approach to detect *Campylobacter* in air samples is based on the detection of characteristic volatile organic compounds (VOC) from chicken faeces of colonized birds in barn air, or from the headspace over poultry faeces^23,24^*.* These methods do not detect the bacteria themselves, but rather their VOC fingerprint as already shown for *C. jejuni*-colonized humans, in which the presence or absence of specific VOCs were associated with the presence of *Campylobacter*^25^. They are based on gas chromatography and subsequent detection of characteristic compounds by mass spectrometry^25^ or metal oxide gas sensors^24^. Like the methods based on air dust, the methods based on VOC detection have a huge potential for automation and could be used for continuous *Campylobacter* vigilance in poultry barns. However, it is not clear how sensitive these methods are in comparison to established methods in controlled settings.

In the present study, our aim was to assess the performance of two air sampling methods (VOC and dust-based) with established methods based on microbial analysis of faecal material. We used a unique, cage-based approach to assess these detection methods on poultry flocks, with pre-defined infection levels to allow correlation of results and a true comparison between the *Campylobacter* detection methods.

## Materials and methods

### Study set-up

The investigation was carried out in two simultaneous experimental runs. A total of 24 birds were supplied as day-old chickens (day 0) taken directly from a commercial hatchery. The birds were divided into two groups with 12 birds each and kept in two separate rooms in a biosafety level 2 animal facility of the Research Centre for Emerging Infections and Zoonoses (RIZ) at the University of Veterinary Medicine Hannover, Foundation, Hannover, Germany. Each room was equipped with its own ventilation system and upstream hygiene lock. The RIZ animal facility harbours a special technology to reduce the pressure in all labs/stables. The air in the rooms was changed nine to 10 times per hour (measured air change rate 9.17 and 9.97, respectively). The relative air humidity amounted to approx. 56% at a room temperature of 22 °C during the experiment. Based on a mean body weight of chickens of 2 kg during the course of the experiment, a room volume of about 148 m^3^ and an air change rate of at least nine times per hour, this resulted in an air volume flow of 55.5 m^3^/kg body weight/h. The birds were kept under a 16-hour light, 8-hour dark lighting schedule, while stocking density never exceeded 30 kg per square metre.

The animals were fed in three phases with conventional complete diets. The diets were analysed by standard procedures in accordance with the official methods of the VDLUFA^26^, as described in a previous study by Visscher et al.^27^. The diet offered during the experiment (day 14 until end of the experiment) contained 19.7% crude protein and 12.5 MJ AME per kg diet.

**Figure 1:**
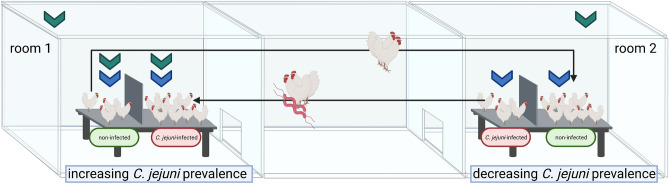
Chickens were kept in two rooms under special biosafety measures in the animal facility. Every three days, two animals from each box changed rooms. The continuous wall prevented direct contact between the artificially *C. jejuni*-infected and non-infected birds kept in one box. Green arrows mark the positions of air sampling for VOCs analysis, which occurred twice each day. Blue arrows mark the positions of electrostatic air sampling for qPCR analysis, which ran once every day. The figure was created with http://biorender.com.

In both rooms, identical boxes were installed, which were divided by a continuous wall, which will later prevent direct contact between artificially *C. jejuni*-infected and non-infected birds. At the start of the experiment, 12 birds were kept exclusively on one side of the box (Fig. 1). Each half of the box was littered with wood shavings (1 kg/m^2^).

At day 17 of life, each of the 12 birds of only one room (room 2, Fig. 1) was administered orally with 1 mL of a *C. jejuni* inoculum (challenge dose of 7.64 log_10_ CFU/mL of challenge inoculum). The *C. jejuni* inoculum was a mixture of a *C.* *jejuni* strain C356 (DSM 24306, Leibniz Institute DSMZ—German Collection of Microorganisms and Cell Cultures, Braunschweig, Germany) and a field strain isolated from chickens participating in previous studies by Hankel, et al.^28^. The inoculum was prepared as described in Hankel, et al.^29^. The conserved strains for experimental inoculation were cultivated at 41.5 °C in a microaerobic atmosphere on solid selective culture media (mCCD agar; Oxoid Germany GmbH, Wesel, Germany). The challenge strains were used in their stationary growth phase (24 to 48 h). An isotonic 0.9% sodium chloride solution was used as the basis for the infection bouillon. A defined density of *C. jejuni* was adjusted (0.5 McFarland units) using a densitometer (DEN 1B, biosan SIA, Riga, Latvia), which already corresponds to the targeted challenge dose. The challenge dose was verified by plating dilutions of bacterial culture and quantifying colonies. Prior to the experimental challenge, birds were confirmed *Campylobacter*-negative via qualitative bacteriological examination. Three days later, the success of the experimental infection was checked via qualitative detection of *C. jejuni* in cloacal swabs of all experimentally infected birds, so that at day 21, the experimental phase started.

**Figure 2:**
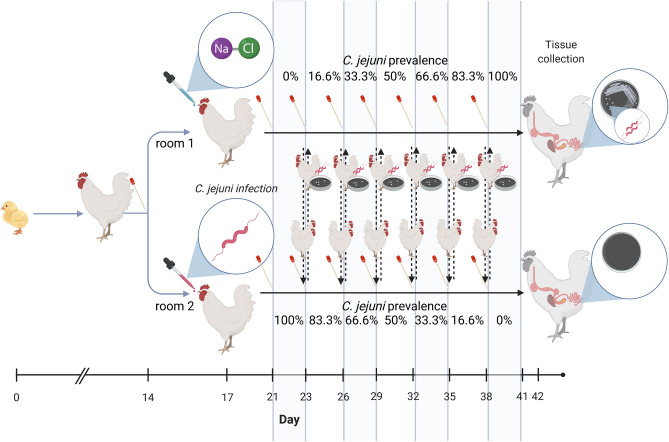
Experimental timeline. At day 14, a total of 24 birds were divided in two groups and kept in two different rooms. All birds prior to the experimental challenge were confirmed *Campylobacter*-negative via qualitative bacteriological examination of cloacal swabs. At the same day of life, day 17, each of the 12 birds kept in room 2 was experimentally challenged with *C. jejuni,* while all birds in room 1 received 1 ml of an isotonic 0.9% sodium chloride solution. Three days later, the success of the experimental infection was checked via qualitative detection of *C. jejuni* in cloacal swabs of all experimentally infected birds, so that at day 21, the experimental phase started and lasted for 21 days. Within these 21 days, a cloacal swab was obtained from each chicken every three days for qualitative detection of *C. jejuni*. Additionally, a quantitative bacteriological examination of *C. jejuni* was performed in excreta samples of the two birds in room 2 that subsequently changed rooms. The experiment ended at day 41 of life. Birds were dissected one day later to obtain caecal contents for qualitative detection of *C. jejuni*. The figure was created with http://biorender.com.

From this time onwards, two non-infected chickens were removed and replaced with two *C. jejuni*-infected chickens in room 1 and vice-versa for room 2 (see Fig. 1). The two birds that had changed rooms were placed in the wider not yet occupied half of each box (*C. jejuni*-infected half in room 1 and non-infected in room 2). The continuous wall prevented direct contact between the non-infected and artificially infected birds when kept in one box during the entire experiment. This guaranteed that each animal was kept once in each room and box and therefore exposed to identical environmental conditions. In addition, increasing and decreasing *C. jejuni* prevalences were adjusted in the rooms in a controlled manner for the investigations. The replacement process, using two chickens each time, was repeated five times during the following 15 days to allow a complete replacement of non-infected with infected birds in room 1 and infected with non-infected birds in room 2. In each room, three days worth of samples were taken after each replacement. This process provided samples from 0% to 100% infected birds in room 1 and 100% to 0% infected birds in room 2 (see Fig. 2).

At day 42, all chickens were culled by cervical dislocation and subsequent exsanguination and afterwards dissected. Qualitative detection of *C. jejuni* was performed in caecal samples of all birds. A timeline of the experiment is shown in Fig. 2.

### Sample collection

Electrostatic air sampling for subsequent qPCR analyses was performed once a day at day 17 (before infection) and from day 21 to day 41 every day in the morning at animal level by using the AeroCollect® system (FORCE Technology, Brøndby, Denmark) for periods lasting approximately 5 min (flowrate: 200 ml/min). All measurements always took place at the same time of day.

Volatile organic compounds (VOCs) were collected using a free-standing air sampler system (RoboScientific Ltd., Littleport, UK) fitted with adsorbent material pads (Cooker Hood Grease & Carbon Filter, Quinnspares (Ezee-Fix), Derry, United Kingdom). The VOCs were collected from the air and pre-concentrated on the adsorbant material to enable subsequent measurement on the 307B VOC Analyser (RoboScientific Ltd.). For this purpose, three permanently installed samplers were set up in room 1 (increasing prevalence). The samplers were arranged to cover the base of the cages and to collect air from the whole of the room by placing them beneath the exhaust vent. Therefore, one sampler was fixed in room 1 directly above the birds in each of the corresponding half of the box (Fig. 1). A third sampler was installed directly under the exit fan in both rooms (Fig. 1).

The samplers were set for an average volume of 92 m^3^/h of air containing the VOCs to pass through the adsorbant pads and were run every day (from day 21 to 41) twice for 60 min. Thereafter, the content was removed and placed in 120 mm × 200 mm metallised grip seal bags and stored frozen at − 20 °C until analysis with the VOC analyser. Fresh sample pads were inserted into the samplers ready for the next sampling run.

Before the birds were moved between the rooms every third day, the *C. jejuni* prevalence was assessed by qualitatively testing cloacal swabs taken from all birds for the presence or absence of *C. jejuni* (day 23, 26, 29, 32, 35, 38, and 41). In addition, quantitative detection of *C. jejuni* in excreta samples of the moving *C. jejuni*-positive birds was performed in order to examine excretion of *C. jejuni*.

### Microbiological analysis

In accordance with § 64 of the German Food and Feed Code (Lebensmittel-, Bedarfsgegenstände- und Futtermittelgesetzbuch, LFGB), the qualitative bacteriological examination was performed following DIN EN ISO 10272-1:2017^30^. Samples were prepared in a one-to-nine ratio in Bolton broth and incubated in a microaerobic atmosphere for 4 h at 37 °C followed by 44 ± 4 h at 41.5 °C. The microaerobic atmosphere was created in a CO_2_ incubator with O_2_ control (oxygen content of 5 ± 2%, carbon dioxide content of 10 ± 3%). After enrichment, samples were streaked onto two solid selective culture media (modified Charcoal Cefoperazone Deoxycholate (mCCD) agar and Karmali agar; Oxoid Germany GmbH, Wesel, Germany), incubated for 44 ± 4 h at 41.5 °C in a microaerobic atmosphere. After incubation, plates were checked for the presence of typical *Campylobacter* colonies (grayish, often with a metallic glow, flat and moist, and prone to spread). Bacterial morphology (curved rods) and motility (spiral corkscrew-like motility) of at least one characteristic individual colony was examined using phase contrast microscopy. Qualitative bacteriological examination of cloacal swabs revealed that the desired prevalence steps were successfully established (Table 1).

**Table 1:** *C. jejuni* prevalence in cloacal swabs as well as in caecal content at day 42.

Day	Room 1	Room 2
	*C. jejuni* prevalence	*C. jejuni* prevalence
21–23	0.00%	100%
24–26	16.6%	83.3%
27–29	33.3%	66.6%
30–32	50.0%	50.0%
33–35	66.6%	33.3%
36–38	83.3%	16.6%
39–41	100%	0.00%
42 (caecal content)	100%	0.00%

For quantitative bacteriological examination, 0.5 g sample material was diluted with phosphate buffered saline in a tenfold dilution series. In duplicate, 100 μL of each dilution was plated onto mCCD agar and incubated in a microaerobic atmosphere for 44 ± 4 h at 41.5 °C. The characteristic colonies were counted and an average value from the two duplicate experiments was taken for calculating the CFU/g intestinal content. To confirm the presence of *Campylobacter*, morphology and motility of five presumptive individual colonies per plate were analysed by phase contrast microscopy. As no confirmation of all of the grown colonies took place, there is a possibility that the number of colonies was overestimated. Continuous excretion of *C.* *jejuni* with the excreta of the birds when moved from room 2 to room 1 was confirmed by quantitative bacteriological examination (Table 2).

**Table 2:** Putative *C. jejuni* counts (log_10_ CFU/g) in excreta samples of *C. jejuni*-positive moved birds.

Day	Bird 1	Bird 2
23	4.22	5.78
26	5.90	3.77
29	5.45	5.59
32	5.62	5.55
35	4.83	5.53
38	6.05	5.41

### qPCR in air samples

The air samples collected in the disposable sample chambers were stored at room temperature until the conclusion of the study and transported under standard shipping conditions. All samples were analysed in the AeroCollect laboratories at FORCE Technology as blinded samples.

The collected material in the disposable sample chambers were eluted by pipetting in 25 µL Mili-Q water. Each sample was analysed in duplicate according to the procedure, primer and probes originally described by Josefsen, et al.^31^. Briefly, 2 µL template from the eluted samples were analysed in a total volume of 20 µL containing 10 µL qPCR master mix (PerfeCTa^®^ qPCR ToughMix^®^, Quanta Biosciences Inc., Beverly, MA, USA) probe, forward and reverse primers at a final concentration of 0.05 µM, 0.44 µM and 0.48 µM, respectively. Water was added to reach the final concentration of 20 µL. The reactions were performed in an Agilent Aria Mx qPCR (Agilent Technologies, Inc., Santa Clara, CA, USA). After 3 min at 95 °C, 40 cycles were performed at 94 °C for 20 s and 58 °C for 60 s, respectively. The detection limit is between 0.3 and 3 genomic copies/µl.

### VOCs analysis

The samples were analysed for the total VOCs collected in the following manner: each metallised bag was heat sealed on the outside edge of the grip seal to prevent leakage. Then an 18G needle was inserted into the bag through a small piece of PVC insulation tape (to prevent the bag wall from splitting) and air was pumped into the bag using a diaphragm pump until it was inflated (200 mL). A second piece of PVC insulation tape was used to seal the hole. The inflated bags were then incubated at 80 °C for 30 min to volatilise the VOCs from the adsorbant pads into the headspace air. Each bag was removed from the 80 °C oven shortly before analysis, then allowed to cool to room temperature (20 °C) for five minutes, then sampled four times using a Model 307B VOC analyser (RoboScientific Ltd.) fitted with 12 organic semi-conducting (OSC) sensors configured into an array that were chosen to be sensitive to the VOCs associated with *Campylobacter* species. These VOCs were based on previous analysis and with reference to the literature and included 1-pentanol, 1-octanol, 1-nonanol, acetoin, dimethyl disulphide and isovaleraldehyde^24^. Data from the sensor array were automatically recorded by the software running the Model 307B. After each analysis run, the sensor array of the VOC analyser was internally cleaned in deionised water using headspace vapour from a 5%v/v solution of butan-2-ol before shutting down the analyser.

The aforementioned VOCs analyses used the following two parameters; the divergence (from the baseline), which is the same as the maximum peak height, and the integrated area under the sensor response curves. The Model 307B VOC Analyser provides two outputs per sensor per parameter. Therefore, for 12 sensors, there are a total of 24 outputs, and with two different parameters, there are 48 data points per sample used to describe the obtained data.

### Statistical analyses

McNemar's test was used to assess the differences on a dichotomous dependent variable (*C. jejuni* status) between the bacteriological examination and electrostatic air samplers. The results of the bacteriological examination were set as gold standard.

The obtained sensor data (VOCs) were processed using Linear discriminant Analysis (LDA) in a statistical package XLStat (Addinsoft, Paris, France). The two parameters used were the divergence (from the baseline), which is the same as the maximum peak height, and the integrated area under the sensor response curves. The Fisher distance was used to measure dissimilarity between sensor data. 2-D Discriminant analysis cluster plots were used to visualise the multi-dimensional sensor data in a 2-D vector space. All statements of statistical significance were based on *p*-values smaller than 0.05.

### Ethics approval and consent to participate

The animal experiments were carried out in accordance with German regulations. The experiments were approved by the Committee on Animal Experiments of the Lower Saxonian State Office for Consumer Protection and Food Safety (Niedersächsisches Landesamt für Verbraucherschutz und Lebensmittelsicherheit [LAVES]; reference: 33.12-42502-04-19/3184). We confirm the study is reported in accordance with ARRIVE guidelines.

## Results

### General animal health

The experiment ran without complications. No animal losses or animal health concerns were recorded. At the end of the 42-day rearing period, the chickens had a body weight of in average 3.015 g (*C.* *jejuni*-negative birds) and 3.140 g (*C.* *jejuni*-positive birds), respectively.

### Environmental samples

#### Electrostatic air sampling

The results of PCR analyses of *C.* *jejuni* are shown in Table 3 and described in the following.

**Table 3:** Threshold cycle (Ct) values of PCR analyses of *C. jejuni* (*C.j.*) in air samples collected at non-infected and *C. jejuni*-infected areas in each room.

Sampling day	17	21	22	23	24	25	26	27	28	29	30	31	32	33	34	35	36	37	38	39	40	41
**Room 1**																						
**Prevalence**	0%	0%			16.6%			33.3%			50.0%			66.6%			83.3%			100%		
**Animal number in box halves**																						
Non-infected	12	12	12	12	10	10	10	8	8	8	6	6	6	4	4	4	2	2	2	0	0	0
*C.j.*-infected	0	0	0	0	2	2	2	4	4	4	6	6	6	8	8	8	10	10	10	12	12	12
**Ct values**																						
Non-infected	–	–	–	–	–	–	–	–	–	–	–	–	–	–	35.4	–	36.3	36.2	37.0	34.4	–	38.0
*C.j.*-infected	–	–	–	–	–	–	–	–	–	–	33.4	30.9	32.5	33.6	31.1	33.5	32.1	32.5	32.5	29.4	32.4	31.1
**Room 2**																						
**Prevalence**	0%	100%			83.3%			66.6%			50.0%			33.3%			16.6%			0%		
**Animal number in box halves**																						
*C.j.*-infected	12^a^	12	12	12	10	10	10	8	8	8	6	6	6	4	4	4	2	2	2	0	0	0
Non-infected	0	0	0	0	2	2	2	4	4	4	6	6	6	8	8	8	10	10	10	12	12	12
**Ct values**																						
*C.j.*-infected	–	30.4	28.7	32.1	34.7	32.3	31.3	36.1	32.7	33.5	37.1	33.5	31.6	–	30.1	31.3	31.2	31.1	37.0	34.6	–	–
Non-infected	–	–	34.2	–	–	–	37.9	–	–	–	–	–	–	37.4	36.4	–	–	–	–	–	–	–

^a^Before experimental infection.

##### Room 1 (increasing prevalence)

From a prevalence of 50% (day 30–32 of the experiment) or from a number of six positive birds, *C. jejuni* was detected in the air samples collected above positive animals in room 1 and on all further days of the experiment (12 days).

##### Room 2 (decreasing prevalence)

On a total of 18 of 21 days, *C. jejuni* was detected in air samples collected above positive birds from the first day of measurement (100% prevalence with 12 animals shedding *C. jejuni*). Once in the middle and on the two last days of the experiment, *C. jejuni* was not detected in air samples taken above positive birds. From time to time, the samples collected over the non-infected birds located closely to *C. jejuni*-infected birds were also *C. jejuni*-positive. To be precise, on 6 days in room 1 and 4 days in room 2.

#### Comparison of electrostatic air samplers and cultivation-based method

The kappa value (Table 4) shows a good agreement in both tests and no significant differences were observed for the percentage of positive evaluated birds (*p* = 0.0703). Nevertheless, electrostatic air samplers diagnosed fewer birds as *C. jejuni*-positive compared to the cultivation-based method. Sensitivity of electrostatic air samplers was 80.1%, while specificity amounted to 83.3%.

**Table 4:** McNemar test and kappa statistics evaluating electrostatic air samplers above the area where *C. jejuni-*infected birds were kept with increasing and decreasing animal numbers.

Electrostatic air samplers	*C. jejuni* status of birds according to cultivation-based method		Row total
	*C. jejuni*-negative	*C. jejuni*-positive	
***C. jejuni*-negative**			
Frequency	5	7	12
Percent	11.90	16.67	28.57
Row Pct	41.67	58.33	
Column Pct	83.33	19.44	
***C. jejuni*-positive**			
Frequency	1	29	30
Percent	2.38	69.05	71.43
Row Pct	3.33	96.67	
Column Pct	16.67	80.56	
**Column total**			
Frequency	6	36	42
Percent	14.29	85.71	100.00

*Pct* Percent, *p*-value of McNemar’s test: 0.0703; kappa value: 0.7534.

#### VOCs results

**Figure 3:**
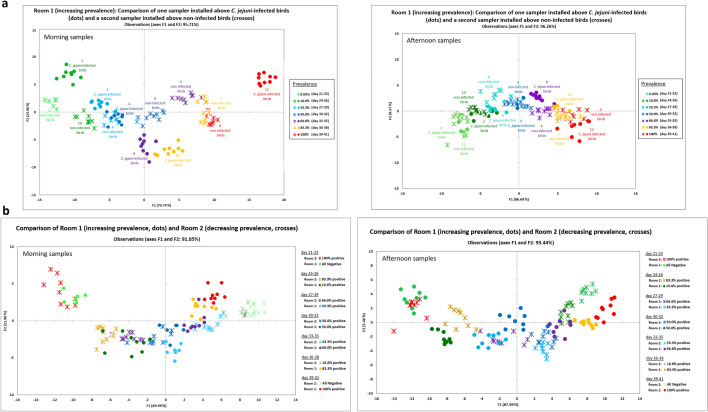
2-D Discriminant analysis plots of VOCs data sampled in the morning and afternoon from (**a**) room 1 (increasing *C. jejuni* prevalence) with one sampler directly installed above *C. jejuni*-infected and one sampler directly installed above non-infected birds, and (**b**) samplers directly installed under the exit fan in room 1 and 2. Each point represents sensor data measured at one time point (VOCs). VOCs were measured three times for one sample. One sample was taken twice a day in the morning and in the afternoon on three consecutive days. Points of the same colour/shape indicate one prevalence step which was maintained over three days.

When comparing VOCs sampled in the morning in room 1 above *C. jejuni*-infected and non-infected birds (Fig. 3a), the sensors in the array were able to distinguish differences in VOCs between infected and non-infected birds at any time during the experiment, while distances between data points increase with increasing number of *C. jejuni*-infected birds and age/progressing days of the experiment. Nevertheless, when comparing VOCs sampled in the afternoon in the same room, a distinction between infected and non-infected birds based on differences in VOCs was less clear compared to samples taken in the morning. Basically, the sensors followed variations in the levels of VOCs associated with progressing days in the experiment. When comparing VOCs sampled directly under the exit fan of room 1 and room 2 (Fig. 3b), the sensors in the array were able to distinguish differences obtained from the age of 36 days onwards between a *C. jejuni* prevalence of 16.6% and 83.3% as well as 0% and 100%. This can be seen in the 2-D discriminant analysis cluster plot of the morning and the afternoon samples (Fig. 3b). However, the data points belonging to the respective prevalence from day 36 of the experiment onwards (16.6% vs. 83.3% and 0% vs. 100%) appear further apart when the samples were taken in the morning compared to the afternoon, which suggests that the level of measured VOCs differ more at this time of the day.

## Discussion

### Non-invasive air sampling to detect C. jejuni status

#### PCR analyses of C. jejuni in air samples

From the results of PCR analyses in air samples, conclusions could be drawn regarding the presence or absence of *C. jejuni*. Compared to culture-based methods, sensitivity of electrostatic air samplers (80.1%) and specificity (83.3%) were lower compared to the findings of real-time PCR in faecal samples^19^. While compared to results obtained by microaerobic culture, there was no statistically significant difference in performance between real-time PCR and culture by selective enrichment, and the diagnostic specificity was 0.96 with an agreement of 0.92 in chicken faecal samples^32^. However, Olsen et al.^21^ described the sensitivity of detection of *Campylobacter* in air as being comparable to that in other sample materials. It can be assumed that, among other reasons, this is a question of DNA concentration. In the present study, *C. jejuni* was reliably detected in air samples from a total of six animals, six days after the first arrival of *C. jejuni*-positive birds. Also in room 2, five days had elapsed after experimental infection of the birds before the first air samples were taken. The question arises whether a five-six-day period is needed for dust from the excrements to develop, which carries DNA of *C. jejuni* that could be measured (detection limit: 0.3 to 3 genomic copies/µl). Similar to the present study, PCR results in sock samples decreased from the detection level of Ct = 40 to a Ct value of 24.04 in 3 days, while the increase in the amount of *Campylobacter* detected in the air and dust was more gradual^33^. However, in the same study, the reverse trend was also seen. *Campylobacter* was first negligibly detected in sock samples 11 days after detection in the air^33^. Also, Søndergaard et al.^22^ concluded from their study findings that via air samples combined with quantitative real-time PCR, *Campylobacter* contamination could be detected earlier than by boot swabs. Besides the time after *C. jejuni* entry, the number of excreting birds and thus probably the amount of DNA present in the air could be a relevant factor, too. This hypothesis can be supported by the results obtained from the experimental set-up in the present study. Compared to recommendations for stable air volume flow of 2.8 m^3^/kg body weight and hour (chickens with a body weight of 2 kg in summer with a temperature < 26 °C^34^), mean air volume flow during the experiment was about 20 times higher (55.5 m^3^/kg body weight and hour) and the apparent lesser sensitivity of the AeroCollect system in the present study was more likely a consequence of study design. This study shows that it is possible in principle to detect a colonized flock, but it is not possible to extrapolate the sensitivity of this method to a commercial flock scenario with the data obtained in the present study. Under practical conditions, both versions are conceivable, an easier or more difficult detection compared to the experimental conditions.

#### VOCs analyses in air samples

The presence of two *C. jejuni*-positive birds in the experimental room alone was sufficient to detect significant differences in the VOCs composition of air samples, when sampled directly above the chickens. Nevertheless, it seems to matter at what time of the day the samples are taken. We were able to show that the time of sampling during the day could play a role in this method and clearer results could be obtained when sampling in the morning. Several environmental factors and sampling conditions significantly influence fecal VOC profiles^35^. For example fecal samples were found to be highly unstable over time when stored at room temperature, mainly due to ongoing bacterial fermentative processes, resulting in distinct metabolic profiles^36^. Broiler chickens exhibit diurnal rhythms for feed intake^37,38^. This activity is negligible during darkness, while feed intake peaks immediately within the first 20 min following scotoperiod^37^. Highest amounts of diet are consumed during the first hour following scotoperiod compared to the other hours of the day^38^. About two hours following scotoperiod, the animals begin to shed fresh excreta^37^. In the present study, the light was turned on three hours before samplers run over a period of one hour, which, in turn, could have lead to a higher abundance of VOCs in the air emitted from a greater amount of fresh excreta during this time of day compared to the afternoon. It should also be noted that VOCs mix rapidly in the air. This could have complicated the differentiation into *C. jejuni*-infected and non-infected birds by the two samplers installed directly above the animals, especially in the afternoon.

From the results of the VOCs analyses, conclusions could be drawn not only about the presence or absence of *C. jejuni*, but also about the status of the infection process, at least to advanced age of the chickens/progressed days of the experiment (from 36 days onwards). Besides duration of storage at room temperature, also fecal sample mass has a significant influence on fecal VOC composition^35^. Amount of birds excreta increase with age and increasing numbers of kept birds. In this experimental setup, we were able to distinguish differences between a *C. jejuni* prevalence of 16.6% and 83.3% as well as 0% and 100% with only 12 birds per room. In a chicken house under practical conditions, where significantly more animals are kept, the differences in VOCs may become even more pronounced and it might be possible to distinguish between even closer prevalence levels. Still, this remains to be verified.

Furthermore, it was also possible under these study conditions to detect decreasing prevalences via the VOC analyses. This makes this method also interesting in terms of verifying the success of implemented measures to reduce *C. jejuni* at the level of primary production.

The question arises whether this method can only be used for a single room and single pass and whether reference values can be established for other flocks and multiple passes within the same stable via the measured composition of the VOCs. The intestinal microbiota of chickens differs considerably between flocks and multiple passages^39^. It remains to be tested whether the VOCs produced by the microbiota also differ between trials. Additionally, the influence of co-infections with other pathogens or antibiotic treatment might interfere with the composition of VOCs. Nevertheless, measuring VOCs appears to be a promising method to monitor for infections in chickens by obtaining early warning signs of such infections occurring. Some early trials of real-sized chicken flocks were sampled for VOCs with successful results using the VOC approach (unpublished data).

Both air sampling methods do not provide any information on *C. jejuni* load in the animal’s intestine, although Ct values of electrostatic air sampling correlated with the number of positive birds and VOCs analyses recognised different *C. jejuni* prevalence. Therefore, it seems that the status of the infection can be assessed by monitoring the course of the infection with both air sampling methods, making these detection tools interesting as this knowledge is important for risk assessment for humans. The extent of contamination shows a positive correlation between the number of *Campylobacter* present in the caecal content and the number of bacteria on the carcasses and meat cuts, and finally, the infection risk for humans^40,41^.

## Conclusions

Both non-invasive air sampling methods are generally able to detect *C.* *jejuni* presence and to distinguish the number of present *C.* *jejuni*-positive birds. Therefore, both techniques are promising tools to facilitate *Campylobacter* surveillance in poultry flocks, however, extensive performance testing and validation are still required.

## Data Availability

All data generated or analysed during this study are included in this published article.
